# Analysis of Pollutant Accumulation in the Invasive Bivalve *Perna viridis*: Current Status in the Colombian Caribbean 2020–2023

**DOI:** 10.3390/toxics13020077

**Published:** 2025-01-22

**Authors:** Skasquia Ucros-Rodríguez, Freddy Araque-Romany, Luis Montero-Mendoza, Vanessa C. Sarmiento-Nater, Oriana M. Calvo-Carrillo, Boris Johnson-Restrepo, Jorge L. Gallego, Patricia Romero-Murillo

**Affiliations:** 1 Semillero de Investigación SINBIOMA, Grupo de investigación GIBEAM, Programa de Biología Marina, Universidad del Sinú Seccional Cartagena, Av. El Bosque Trans, 54 N° 30-453 Santillana, Cartagena de Indias 130014, Colombia; skasquia_123@hotmail.com (S.U.-R.); freddyarturo0106@gmail.com (F.A.-R.); 2 Environmental Chemistry Research Group, School of Exact and Natural Sciences, San Pablo University Campus, University of Cartagena, Cartagena de Indias 130015, Colombia; lmonterom@unicartagena.edu.co (L.M.-M.); vsarmienton@unicartagena.edu.co (V.C.S.-N.); ocalvoc@unicartagena.edu.co (O.M.C.-C.); bjohnsonr@unicartagena.edu.co (B.J.-R.); 3 Biodiversity, Biotechnology and Bioengineering Research Group GRINBIO, Department of Engineering, University of Medellin, St 87 N° 30-65, Medellín 050026, Colombia; jlgallego@udemedellin.edu.co; 4 Grupo de Investigación GIBEAM, Programa de Biología Marina, Universidad del Sinú Seccional Cartagena, Av. El Bosque Trans, 54 N° 30-453 Santillana, Cartagena de Indias 130014, Colombia

**Keywords:** bioaccumulation, biomonitoring, invasive, marine pollution, trace metals

## Abstract

The Colombian Caribbean faces environmental challenges due to urbanization, industrialization, and maritime activities, which introduce pollutants such as heavy metals, hydrocarbons, and microplastics into aquatic ecosystems. *Perna viridis* (Asian green mussel), an invasive species that has been established in Cartagena Bay since 2009, exhibits potential bioaccumulation capacity, making it a promising biomonitor. This study assessed the concentrations of mercury (Hg), cadmium (Cd), lead (Pb), and selenium (Se) in *P. viridis* across two key sites—a port area at the Cartagena Bay (CB) and Virgen marsh (VM) in Colombia—from 2020 to 2023. Seasonal variations driven by La Niña and El Niño phenomena significantly influenced metal concentrations, with transitional periods modulating pollutant accumulation. The levels of trace metals in soft tissue of *P. viridis* (dry weight) ranged from 0.0003 to 0.0039 µg/g (Cd), 0.04 to 0.21 µg/g (Hg), 0.05 to 1.18 µg/g (Pb), and 0.0029 to 0.0103 µg/g (Se). In suspended particulate matter (SPM), Cd ranged from 0.07 to 0.33 µg/g; Pb ranged from 4.94 to 25.66 µg/g; and Hg ranged from 0.18 to 1.20 µg/g. Results revealed differences in metal concentrations between sites and seasons, highlighting the role of environmental and anthropogenic factors in pollutant distribution. The findings confirm *P. viridis* as an effective biomonitor of complex pollution scenarios in Cartagena Bay. However, its invasive status highlights ecological risks to be addressed, such as interaction with native bivalves and benthic community structures. These results emphasize the need for ongoing monitoring efforts to mitigate pollution and preserve marine biodiversity in the Colombian Caribbean.

## 1. Introduction

The presence of pollutants in coastal areas has significantly increased in recent decades, driven by human settlements, industrial expansion, and agricultural activities [1]. Waste generated from these activities has led to the deterioration of coastal and estuarine ecosystems, particularly in regions with insufficient sewage treatment systems. Coastal pollution is further exacerbated by uncontrolled mining, urban effluents, rural runoff, and the contributions of tourist and industrial vessels [2]. It has been established that these activities generate a series of contaminants that can alter marine fauna, especially those residing directly in affected areas [3]. Various native and non-native faunal groups inhabit these coastal areas, and their impact varies based on their origin and tolerance to environmental conditions and pollutants [4].

Bivalves, a group of sessile, filter-feeding organisms, are distributed worldwide along coastlines and hold significant commercial importance, with many species cultivated globally [5]. Due to their unique biological characteristics and sedentary lifestyle, bivalves can filter large volumes of water and are highly susceptible to accumulating contaminants present in their surrounding environment, including heavy metals, organic compounds, and microplastics [6,7]. This process related to the uptake of contaminants from the environment and its concentration in the organism as the net result of influx (uptake) and efflux is known as Bioaccumulation [8]. Thus, their ability to concentrate chemicals within their tissues provides a tool for monitoring environmental pollution. Mussels and oysters, widely recognized as effective bioaccumulators of environmental pollutants, have been used to assess the quality of the marine environment through monitoring networks like the Mussell Watch Project [9], which promotes research on specific species and pollutants in different regions [10,11].

One of the primary reasons bivalves are so effective as bioindicators is their capacity to reflect the bioavailability of contaminants. For instance, some contaminants may exist in more bioavailable forms when they are dissolved in water, while others may be more readily absorbed from sediment [12]. Factors such as contamination sources, exposure duration, and seasonal changes influence contaminant bioavailability and accumulation in bivalves. Pollutants enter aquatic systems through industrial discharge, agricultural runoff, and atmospheric deposition, with mussels often employed in monitoring due to their global distribution, enabling broad geographic comparisons [13].

The Asian green mussel *Perna viridis* (Linnaeus, 1758) is a potential biomonitor. This bivalve meets key criteria such as wide distribution, a sessile lifestyle, year-round abundance, and high bioaccumulation capacity, which correlate with environmental fluctuations and the presence of contaminants [14,15]. Moreover, in several regions, this bivalve is an invasive species that thrives due to its broad tolerance to salinity and temperature variations [16,17]. In addition, it is commercially valued for its high production of soft tissue compared to other bivalves [18,19]. This bivalve was localized to Cartagena Bay starting in 2009, originating from ballast water [20].

Cartagena Bay has recently been reported as an aquatic environment with ideal characteristics for river and maritime passage due to its natural features and location. It hosts one of the main industrial and tourist centers, making it a critical connection point in the Colombian Caribbean [21]. These activities have led to significant deterioration in the environmental quality of Cartagena Bay due to various sources of pollution, including shipyards, food processors, pesticide and cement factories, the largest crude oil refinery in the country, and multiple loading docks for toxic products such as coal [22,23].

Trace metals contamination is a pressing environmental issue due to these metals’ toxicity, multiple anthropogenic sources, and complex behavior in ecosystems, which pose significant risks to both terrestrial and aquatic life forms, as well as to human health [24,25,26,27,28]. Mercury, resulting from a variety of activities including mining, industrial discharge, port operations, and diffuse pollution, is transported to coastal areas and is distributed across sediments, the water column, biota, and particulate matter [29,30,31]. The association of contaminants with organic matter has resulted in sediments in Cartagena Bay being characterized by significant mercury concentrations in sediments between 0.02 and 0.84 µgHg/g dw, becoming a source of secondary contamination across different trophic levels [21,32]. Other highly toxic metals have also been recorded in sediments, such as Cd ranging from 0.11 to 2.1 µg/g dw and Pb ranging from 3.6 to 54.4 µg/g dw, resulting from industrial and agricultural activities [23].

The assessment of metal concentrations in native and non-native bivalves from the Colombian Caribbean, particularly at Cartagena, remains limited. Existing studies have primarily focused on the native species *Crassostrea rhizophorae*, examining metals such as Ag, Al, As, Cd, Cr, Cu, Hg, Ni, Pb, Ti, V, and Zn [33,34]. Other reports include studies on *Isognomon alatus* (1980, 2009) and *Donax denticulatus* (2012–2013), which evaluated concentrations of Cd, Cu, Hg, Pb, and Zn; and for non-native species, only *Saccostrea* sp. has been studied, with As, Cd, Cr, Cu, Hg, Pb, Ni, Sn, and Zn analyzed during the 2012–2013 period [1,23,35,36]. This study aimed to analyze the role of the non-native mussel *Perna viridis* as a potential biomonitoring organism in Colombia. The investigation initially focused on a bibliographic review highlighting the importance of this species in its native range for monitoring areas impacted by pollutant inputs. Secondly, it centered on research conducted in Cartagena Bay, Colombia, aiming to evaluate concentrations of metals in *Perna viridis* and suspended particulate matter between 2020 and 2023.

## 2. Ecological Aspects of *Perna* spp. in the Caribbean

Invasive organisms or non-native species are those that have been removed from their original environment through human intervention, either voluntarily or involuntarily, and establish themselves in ecosystems that offer significant advantages for their dispersal over native species [37]. Globally, non-native marine organisms are a growing phenomenon due to various factors that facilitate the establishment of these invasive species into ecosystems. These factors include the voluntary transfer of individuals for commercial purposes, such as the development of aquariums or living museums, the transportation of larvae via fishing equipment, or the discharge of ballast water, among others [38]. In the case of bivalves, particularly mussels, there is a frequent association between the introduction of several invasive species within this group and maritime transport through ballast water discharge at ports, as well as interest in aquaculture for species with potential for human consumption [39]. Consequently, non-native bivalves are considered a significant factor in the displacement of native species worldwide, directly impacting the composition of populations and communities of species that inhabit substrates favorable to the establishment of invasive species [16].

In the Colombian Caribbean, non-native bivalve species have been detected in marine environments. Among these, *Electroma* sp., *Corbicula fluminea*, *Perna perna*, *P. viridis*, and *Mytella charruana* have been recorded [20,40]. Among these bivalves, mussels (Mytilidae) are of high commercial interest and are particularly abundant in this region; this is especially true of species belonging to the genus *Perna*, namely *P. viridis* and *P. perna*. These two species exhibit few external morphological traits that distinguish them, sharing high similarity in shell color and shape. In *P. viridis*, shell color ranges from dark brown to greenish-brown, with juveniles displaying a bright green coloration [40,41]. Internally, two key differences have been identified that allow them to be distinguished. In *P. viridis*, the pallial line is more undulated or S-shaped due to the posterior adductor muscle scars extending beyond the pallial line. Additionally, the mantle papillae in *P. viridis* are less pronounced, with a smooth mantle lacking extensions [41]. At the molecular level, this species is differentiated from others within the genus because it has 30 diploid chromosomes instead of 28 [40].

*P. viridis* is native to the Indo-Pacific Ocean and has a wide distribution in the tropical and subtropical zones of this region [15,41]. The introduction of *P. viridis* has been confirmed through various morphological and genetic–molecular analyses, which determined that this species arrived via ballast water or attached to ship hulls from the Indo-Pacific to Trinidad and Tobago. These studies also revealed that its populations exhibit lower genetic variability compared to native populations [39,42]. The first record of this invasive species was documented in Trinidad in 1990, followed by its detection in Florida in 1999. Since then, its presence has been reported in Venezuela (2002), Jamaica (2000–2001), and Georgia, Florida, USA (2002) [43]. Molecular analyses have also confirmed this species’ establishment and persistence in Brazil [42]. In Colombia, the presence of *P. viridis* was initially reported in 2009 in the port area of Cartagena [20], followed by reports from Cispatá Bay in 2010–2011 [44] and Puerto Velero and the Isla de Salamanca National Park in 2018 [45]. The research detailed in this study also noted the presence of this species in the Virgen marsh, specifically in the Bocana sector, complementing the findings from Cartagena in 2009. These findings indicate that this species is likely to be found in other areas of the Colombian Caribbean that remain undocumented.

*P. viridis* is a mussel that is commercially important in this country and is informally distributed to seafood restaurants. In addition, it is considered one of the invasive species of significance in the Caribbean, since it constitutes a threat to native species. This is attributed to its reproductive strategy, rapid development, planktonic dispersal, and strong ability to establish itself in coastal areas [43].

This mussel is typically found in the intertidal zones of sheltered bays and estuaries. Additionally, it has been observed to colonize hard structures, forming dense colonies suspended at depths of up to approximately 20 m [19]. Moreover, this species exhibits a tolerance range of 18–33 salinity and temperatures between 11 and 32 °C. As a filter-feeding organism, it demonstrates high filtration efficiency, ingesting a wide range of suspended organic and inorganic particles from the water column as part of its diet [46].

## 3. *Perna* spp. in Assessing Aquatic Pollution and Environmental Stressors

*Perna* spp., including *Perna viridis* and *Perna perna*, are extensively utilized in ecotoxicological studies for monitoring trace metal pollution in aquatic ecosystems. Their sedentary lifestyle and efficient filter-feeding behavior result in the bioaccumulation of pollutants at concentrations significantly higher than those in their surrounding environment [20,47]. The effective response of these species to environmental contaminants, especially metals, along with their life cycle characteristics, abundance, ease of collection, and sufficient tissue availability, are important criteria that support their suitability as biomonitor candidates [48,49].

For example, research conducted in the Vellar Estuary, India, demonstrated that *P. viridis* bioaccumulates metals such as copper, zinc, and lead, with concentrations reaching up to 25 µg/g dry weight for copper [50]. This accumulation was directly linked to industrial discharges, reflecting local pollution. Similarly, in Hong Kong, *P. viridis* was deployed in seasonal biomonitoring programs, detecting significant levels of cadmium and lead, particularly in urbanized areas such as Victoria Harbour [51,52]. Over the decades, a decline in pollutant levels was observed, correlating with pollution abatement initiatives like the Harbour Area Treatment Scheme.

In Malaysia, *P. viridis* has been similarly applied to assess contamination in various environments. In the Pasir Gudang coastal area, concentrations of lead and cadmium in mussels were reported to exceed permissible limits, with lead levels reaching 6.55 µg/g [53]. The contamination was traced to industrial and maritime activities near aquaculture zones, raising concerns about the consumption of mussels from polluted areas. In the Ennore Estuary, India, *P. viridis* accumulated substantial amounts of iron, manganese, and zinc in its tissues, which were linked to discharges from thermal power plants [47]. These findings were complemented by observations of histopathological damage in the mussels, such as thickened digestive epithelia and hemocytic infiltration in gills, emphasizing their sensitivity to metal-induced stress.

The utility of *Perna* spp. in global pollution monitoring has been further established through large-scale initiatives like the Mussel Watch Program [15]. This program pioneered the use of bivalves, including *P. viridis* and *P. perna*, to monitor trace metals and organic pollutants, such as organochlorines and tributyltin, across regions including Thailand, India, and the Philippines. In areas of high maritime activity, mussels exhibited significant contamination by tributyltin from antifouling paints and elevated levels of organochlorines like DDT and hexachlorocyclohexane, reflecting ongoing agricultural and maritime pollution practices despite international restrictions. Similarly, research along the southeastern coast of China demonstrated the capacity of *P. viridis* to monitor cadmium, lead, and mercury contamination, particularly in coastal regions impacted by rapid industrialization [54].

The biological traits of *Perna* spp. make them highly effective in reflecting spatial and temporal pollutant gradients across diverse environments. Studies in Japan have shown that *P. viridis* can effectively map variations in polycyclic aromatic hydrocarbons (PAHs) and heavy metals in urbanized coastal areas, underscoring their adaptability and reliability as biomonitors [51]. Furthermore, investigations along Malaysia’s coast revealed that *P. viridis* accumulates significant levels of PAHs, particularly in areas influenced by industrial and vehicular activities, offering valuable insights into the sources of petroleum-based hydrocarbon pollution [55]. Beyond metals and hydrocarbons, *P. viridis* and *P. perna* have also been shown to bioaccumulate microplastics and associated toxic metals like arsenic and nickel. This capacity to track emerging contaminants, combined with their demonstrated ingestion efficiency linked to size and filtration dynamics, expands their relevance in modern ecotoxicological assessments [7].

In addition to current pollution monitoring, *Perna* spp. also offer insights into historical contamination trends. Studies in Thailand utilizing *P. viridis* shells indicated their ability to accumulate trace metals such as chromium, manganese, and zinc over time, making them useful for reconstructing long-term environmental changes [56]. Similarly, in the Johore Straits of Malaysia, analysis of specific soft tissues, such as gills and gonads, provided detailed views of metal bioavailability and contamination patterns. Zinc levels as high as 167 µg/g were recorded in gills at industrially influenced sites, further validating the tissue-specific approach to biomonitoring [57]. Another study in Peninsular Malaysia documented bioaccumulation of cadmium, copper, lead, and zinc in *P. viridis*, with pollution indices correlating these levels to anthropogenic activities like aquaculture and port operations [57].

The Caribbean region faces unique environmental challenges, including heavy maritime traffic, extensive industrial activities, urbanization, and reliance on agriculture, all of which contribute to significant pollution loads in coastal and marine ecosystems. The region’s economic dependence on fisheries, tourism, and aquaculture underscores the critical need for effective environmental monitoring to protect biodiversity and sustain local economies. Studies have highlighted that industrial and urban wastewater inputs, coupled with maritime activities, are major contributors to heavy metal and hydrocarbon contamination in Caribbean waters [58,59].

As sessile filter feeders, *Perna* spp. accumulate pollutants directly from the water column and sediment, integrating contaminant levels over time and across various sources. This makes them exceptional bioindicators for assessing complex pollutant interactions in diverse environmental conditions. For instance, in regions like Trinidad’s Gulf of Paria and Jamaica’s Kingston Harbour, *Perna* spp. have been used to track heavy metals such as cadmium, lead, and chromium, providing critical data on industrial and urban impacts on coastal waters [58,59]. Furthermore, studies in Cienfuegos Bay, Cuba, utilizing *P. viridis*, have highlighted their role in detecting hydrocarbons and organochlorine pesticides, offering a comprehensive perspective on pollutant sources and dynamics. This suggests the need for a holistic approach to assess and mitigate pollution impacts using bioindicators [60]. Biomonitoring using *Perna* spp. reveals pollutant distribution patterns, identifies contamination hotspots, and assesses pollution mitigation efforts, highlighting their utility in tracking heavy metals, hydrocarbons, and organochlorines across Caribbean sites like Trinidad and Jamaica to inform ecological management and conservation strategies [58,59,60,61].

## 4. Materials and Methods

### 4.1. Study Area

Cartagena Bay, located on the northern Caribbean coast of Colombia, is of significant importance to tourism, industry, and ports [62]. It is located at the coordinates 10°16′ N and 75°36′ W [32]. This water body is a semi-enclosed system with two openings (Bocagrande and Bocachica) that allow direct communication with the Caribbean Sea. This promotes periodic water renewal due to wind and current-driven circulation [63]. The Bay covers approximately 83 km^2^ with an average depth of 19 m [21] and experiences climatic conditions characterized by three seasons (dry, rainy, and transitional) [64]. The annual ambient temperature ranges between 26.8 and 32 °C, with a surface salinity of 30 to 34 and multi-year precipitation of 1087 mm [23,63].

### 4.2. Sampling Sites and Sample Collection

**Figure 1:**
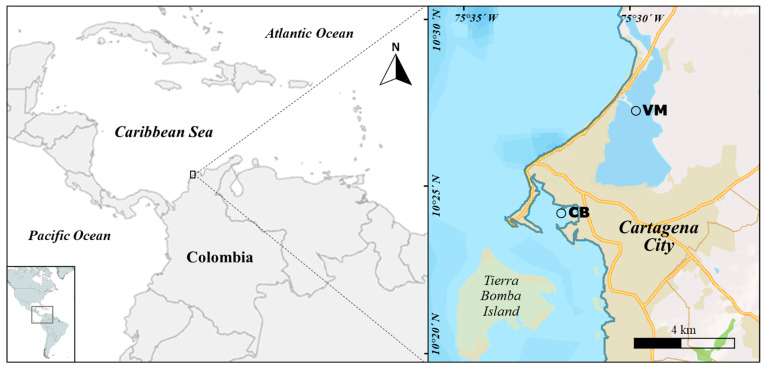
General location and sampling sites CB and VM.

Sampling sites were selected in two environmentally significant locations. The first was in Cartagena Bay, in the port area (CB) (10.394584° N, 75.546856° W), which is a key area for maritime transport, accommodating large cargo vessels, cruise ships, and local fishing and tourism boats. The second site, the Virgen marsh (VM) (10.27108° N, 75.30487° W), is a vital coastal wetland receiving a significant portion of the water from Cartagena city and surrounding areas. These sampling sites are located in the intertidal or shallow subtidal zones (Figure 1). A total of 263 individuals of *P. viridis* were collected per sampling site during the years 2020, 2022, and 2023. These individuals had an average length of 77.9 ± 24.73 mm in 2020, 91.1 ± 16.45 mm in 2022 and 81.3 ± 18.41 mm in 2023. The bivalves were packed in properly labeled zip-lock plastic bags and preserved on ice and stored at a cold temperature (~4 °C). Upon arrival at the laboratory, the shells were cleaned and washed with deionized water. Each individual was measured and weighed, then opened to extract the soft tissue. The tissue was subsequently weighed and stored in plastic containers at −20 °C until analysis. At each location, water samples were collected and filtered through 0.45 μm glass-fiber filters, which were preserved for suspended particulate matter analysis.

### 4.3. Chemicals and Reagents

All reagents were of analytical reagent-grade unless otherwise specified. Ultrapure water with a final resistivity of 18.2 MΩcm^−1^ was used. Nitric acid 65% (HNO_3_), Triton^®^ X-100 (Octil-fenil-polietoxietanol; (C_8_H_17_OH_4_(OCH_2_CH_2_)nOH)), hydrogen peroxide 30% (H_2_O_2_), magnesium nitrate hexahydrate, Mg(NO_3_)_2_·6H_2_O, ammonium dihydrogen phosphate, (NH_4_)H_2_PO_4_, nickel (II) nitrate hexahydrate, and Ni(NO_3_)_2_6H_2_O, were purchased from Merck^®^ (Darmstadt, Germany). The stock standard solutions of Cd (1000 mg/L), Pb (1000 mg/L), and Se (1000 mg/L) were purchased from Merck^®^ (Darmstadt, Germany). The working standard solutions for the calibration curve were prepared daily by serial dilution of the stock solutions of Cd (1000 mg/L), Pb (1000 mg/L), and Se (1000 mg/L), with the addition of a 0.2% *v*/*v* nitric acid solution as the diluent. For the determination of Cd, Pb, and Se in mussel tissues using AAS-GFA, specific matrix modifiers were employed to enhance the accuracy and precision of the method. The selected matrix modifiers were magnesium nitrate Mg(NO_3_)_2_ for Cd, ammonium dihydrogen phosphate (NH_4_)H_2_PO_4_ for Pb, and nickel nitrate Ni(NO_3_)_2_ for Se. The certified reference materials (CRMs) of SRM 2976 (mussel tissues) were provided by the National Institute of Standards and Technology (NIST) of (Gaithersburg, Maryland, USA), DORM-4 (fish protein) was provided by the National Research Council Canada of Canada (Ottawa, Ontario, Canada), and IAEA-457 (marine sediment) was purchased from the International Atomic Energy Agency (Vienna, Austria).

### 4.4. Samples Preparation

Mussel samples, blanks, suspended particulate matter, and standard reference material (SRM 2976) were digested using a MARS 6 microwave digestion system with Xpress Plus vessels (CEM Corporation, Matthews, NC, USA). Mussel samples were freeze-dried to a constant weight using a freeze dryer (model BK-FD10PT, Biobase, SJinan, China), then homogenized. The dried samples were weighed to 500 mg for analysis. Analogously, subsamples of the dry filters were used for suspended particulate matter analysis. Concentrated HNO_3_ (5 mL) and 30% *v*/*v* H_2_O_2_ (2 mL) were added to each vessel, and the mixture was swirled gently. After 15 min, the vessels were closed and placed in a microwave digestion system cavity. The samples were subjected to a digestion program at the maximum power of the microwave (1030–1800 W) and pressure (800 psi) in two steps: (1) the temperature was increased to 200 °C for 25 min; (2) the temperature was maintained at 200 °C for 15 min. The system was cooled down for 10 min to room temperature. Upon completion of the run, the digested solutions were quantitatively transferred and diluted to a final volume of 25 mL with HNO_3_ (0.2% *w*/*v*). Analytical blanks were always run during digestion batches in case of cross contamination. Prior to the experiments, all Xpress vessels were cleaned by soaking overnight in freshly prepared 10% (*w*/*v*) HNO_3_ solution, followed by thorough rinsing with ultrapure water.

### 4.5. Instrumental Analysis

The analytical methods used to measure the amount of Cd, Pb, and Se in *Perna viridis* mussel and Cd and Pb in suspended particulate matter (SPM) samples were based on an iCE 3500 series Atomic Absorption Spectrometer (AAS) equipped with a graphite furnace atomizer (GFA), Zeeman effect background correction and autosampler (Thermo Fisher Scientific, Cambridge, UK). All samples were analyzed in duplicate. The carrier gas was nitrogen, the injection volume was 20 μL, and the area under the curve value was used to determine the measurements and metal concentrations. Pyrolytic graphite-coated tubes were used for all determinations. The concentrations of Cd, Pb, and Se were determined using an AAS-GFA equipped with Zeeman background correction. To enhance the accuracy of trace element determination, calibration curve standards were prepared using a mussel tissue matrix for each element. The calibration standard concentrations were prepared by dilution series and by adjusting the final volume with 0.2% nitric acid solution. For the analysis, 100 µL of standard solutions or digested sample solutions were diluted (1:10) with 900 µL of matrix-modifying aqueous solution containing 0.2% *v*/*v* nitric acid (97%), 10% *v*/*v* Triton X-100 (2.5%), and 20% *w*/*v* magnesium nitrate (0.5%) as a matrix modifier for cadmium. For Pb, the matrix modifier was 20% *w*/*v* ammonium dihydrogen phosphate, and for Se, it was 20% *w*/*v* nickel (II) nitrate. Detailed instrumental parameters for the AAS-GFA used in the analysis are provided in Table 1 and Table 2.

For Hg analysis, the concentrations in mussel samples and subsamples of the dry filter with the suspended particulate matter were determined using a thermal decomposition–atomic absorption spectrometer (TD-ASS) with a Zeeman-effect background correction technique, RA-915M equipped with a pyrolysis accessory, PYRO 915+, for solid sample analysis (Lumex Instruments, St. Petersburg, Russia). The mercury concentrations were measured directly in the dry samples without any sample treatment. Samples were weighed (~100 mg) into a quartz boat and heated at 800 °C to complete combustion in order to release the Hg gas, which was measured based on the absorbance at a wavelength of 253.7 nm in the enclosed system. After thermal release, the quantitative signal for Hg was shown as the total area under the peak. The quantification of Hg was carried out by peak integration using RAPID software. Before every measurement, the quartz boat was cleaned and heated to obtain the instrument baseline. The calibration curve was created using certified standards material. Calibration curves were considered optimal if the regression coefficient (R) value was equal to or greater than 0.995. Method accuracy was quantified using blank analyses and certified reference materials NIST SRM 2976 (mussel tissue) and IAEA-457 (marine sediments).

### 4.6. Quality Control and Quality Assurance

All the plastic and glassware were soaked in nitric acid solution (10% *v*/*v*) for 24 h to be cleaned before the analysis and then rinsed with deionized water before use. The accuracy, precision, linearity, method detection limit (MDL) and method quantitation limit (MQL), range, and linearity were performed to validate this method for determining the concentrations of Cd, Pb, Se, and Hg in *Perna viridis* mussels. For all samples, analyses were performed in duplicate and two certificate reference materials (CRMs), SRM-2976 and DORM-4, were analyzed for quality control purposes. The MDL and MQL were derived from blank measurements. The MDL was calculated as the mean concentration of the digested blanks plus three times their standard deviation (SD), while the MQL was calculated as the mean concentration of the digested blanks plus ten times their standard deviation (SD). The MDL and MQL values were 0.011 and 0.702 μg/g for Cd; 0.547 and 1.12 μg/g for Pb; 3.26 and 7.94 μg/g for Se; 0.151 and 0.420 for Hg. The recovery (%) and relative standard deviation (RSD)(%) were calculated for the CRMs. The results of the SRM-2976 were (mean ± SD, % recovery, %RSD): 1.48 ± 0.127 μg/g, 80.4%, 8.61% for Pb; 0.8 0 ± 0.056 μg/g, 98.0%, 7.0% for Cd; 1.7 ± 0.10 μg/g, 94.1%, 6.0% for Se; 62.1 ± 0.071 μg/Kg, 101.7%, 0.114% for Hg. The following MQLs were obtained for SPM analysis based on a previously developed method [28]: 0.05 μg/g for Cd, 0.0036 μg/g for Hg, and 0.4 μg/g for Pb. The CRM IAEA-457 (marine sediments) yielded recoveries of 108.30 ± 3.1% for Cd, 105.4 ± 3.8% for Hg, and 102.65 ± 4.2% for Pb. The calibration curves were considered optimal if the regression coefficient (r) was ≥0.995.

### 4.7. Statistical Analyses

The concentrations of Cd, Hg, Pb, and Se in *P. viridis* and Cd, Hg and Pb in SPM were analyzed to determine spatial and temporal differences between sampling sites CB and VM across rainy, transitional, and dry seasons from 2020 to 2023. Given that the data did not meet the assumptions of normality or homogeneity, as indicated by the Lilliefors (Kolmogorov–Smirnov) and Bartlett’s tests (*p* < 0.05), nonparametric statistical methods were employed. The Kruskal–Wallis test was used to evaluate significant differences among groups, followed by Dunn’s post hoc test with Bonferroni correction to identify specific group pairs. All statistical analysis were performed using R 4.4.2 statistical software (https://www.R-project.org, accessed on 9 December 2024) and RStudio 12.0 (http://www.rstudio.com, accessed on 9 December 2024). For all comparisons, a value of *p* < 0.05 was considered significant. The results were visualized using bar plots to represent the mean concentrations and standard deviations of each element by site and season, as well as box plots to illustrate data dispersion, distribution, and potential outliers. These analyses provided insights into the accumulation patterns and the spatial and temporal variability of the metals and metalloids studied.

## 5. Results

### 5.1. Cd, Pb, and Hg Concentrations of Suspended Particulate Matter

**Figure 2:**
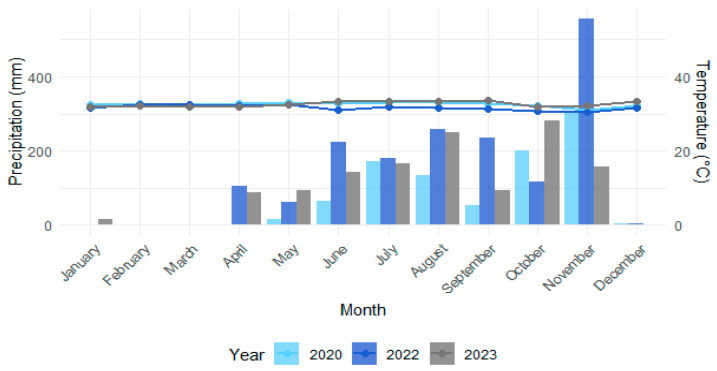
Monthly total precipitation (column charts) and average temperature data (line charts) for the sampling months in the city of Cartagena during 2020 to 2023 were obtained from the DHIME tool of IDEAM (Colombian Institute of Hydrology, Meteorology and Environmental Studies) from the Rafael Núñez Airport station [14015080] (http://dhime.ideam.gov.co/, accessed on 8 October 2024).

The studies conducted during 2020, 2022, and 2023 were influenced by significant changes in rainfall patterns. These changes were driven by the presence of the La Niña phenomenon from 2020 to 2022, which resulted in rainfall levels exceeding the annual average and persisted into early 2023. In contrast, the El Niño phenomenon in 2023 caused a notable reduction in precipitation, particularly toward the end of the year, compared to the previous year (Figure 2) [65].

The concentrations of Cd, Pb, and Hg in SPM presented seasonal variations over the period from 2020 to 2023 (Table 3). Cd levels ranged from 0.07 to 0.33 µg/g, with higher concentrations observed during the rainy season of 2020. Pb had broader variation, with levels at VM ranging from 7.23 to 25.66 µg/g during the rainy seasons and lower during the dry and transition periods. The Hg concentrations were typically lower, from below the limit of detection up to 1.20 µg/g, with a decrease by the rainy season of 2023.

### 5.2. Cd, Pb, Hg and Se Concentrations in Soft Tissue of P. viridis

**Figure 3:**
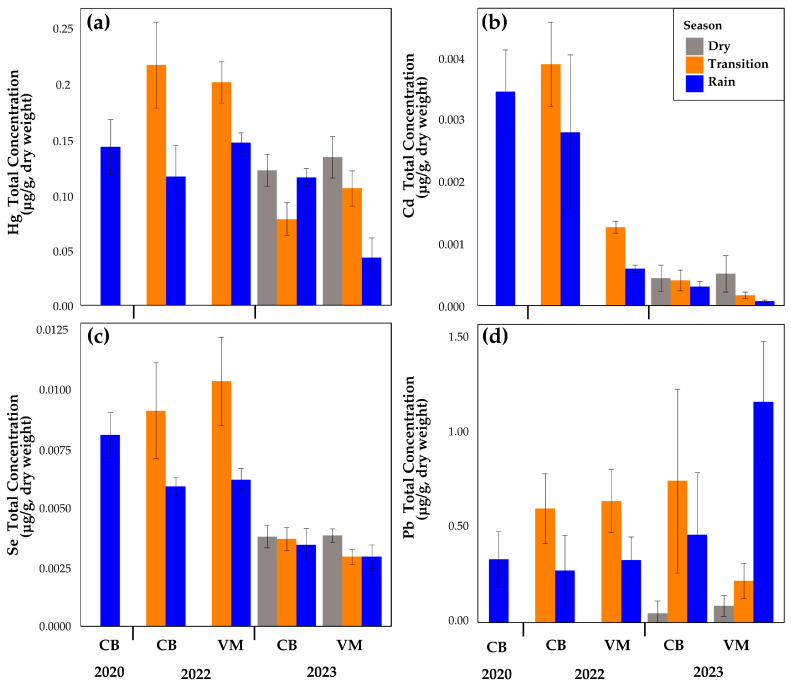
Mean concentrations (µg/g d.w.) and standard deviations of (**a**) Hg, (**b**) Cd, (**c**) Se, and (**d**) Pb in the soft tissue of *Perna viridis* from Cartagena Bay (CB) and Virgen marsh (VM) during the years 2020, 2022, and 2023.

**Figure 4:**
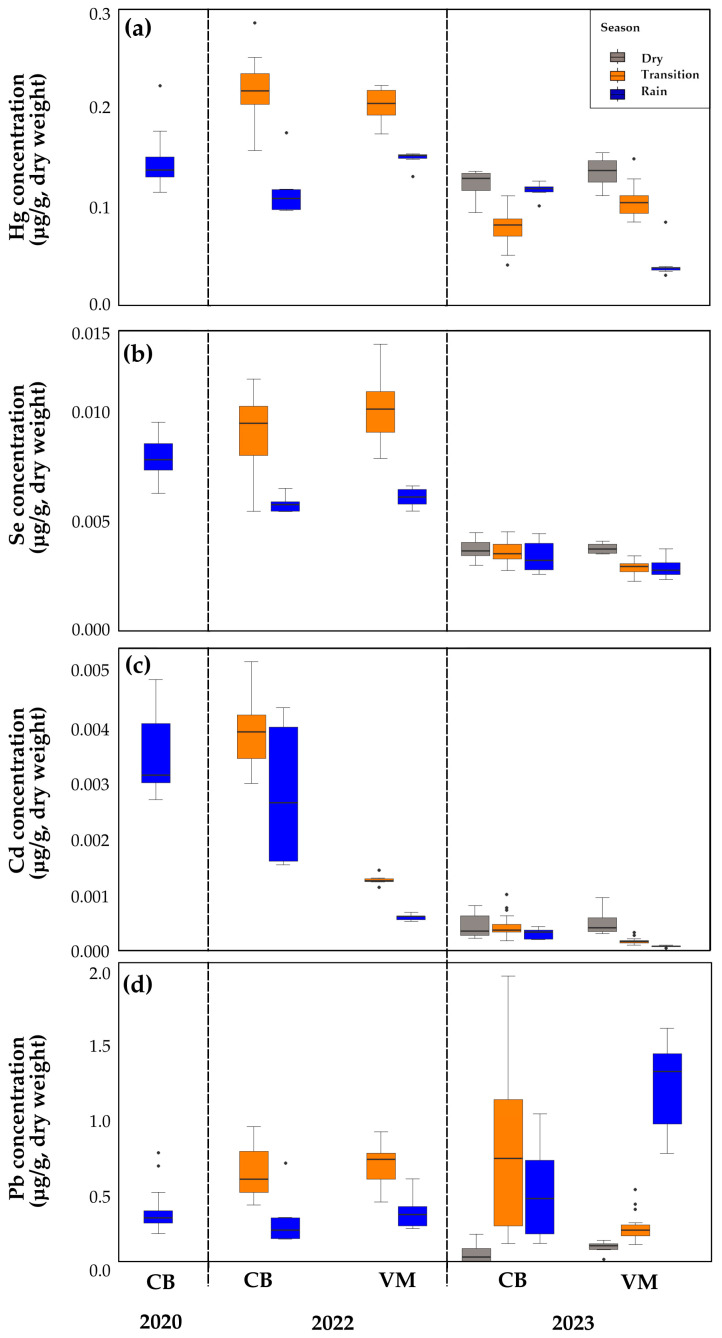
Box-and-whisker plots illustrating the concentrations of (**a**) Hg, (**b**) Se, (**c**) Cd, and (**d**) Pb in the soft tissue of *Perna viridis*. These plots provide a visual representation of the variability in metal and metalloid concentrations Cartagena Bay (CB) and Virgen marsh (VM) during the study period.

Table 4 provides a summary of the mean values and standard deviations for Cd, Pb, Se, and Hg. Figure 3 illustrates the behavior of the elements studied through bar charts. The box-and-whisker plot provides a visual representation of the extent of variability in concentrations across sites and sampling periods (Figure 4).

For Hg, the highest average concentration was observed at CB -22-T (0.1383 ± 0.0255 µg/g), while the lowest average concentration was recorded at VM-23-R (0.027 ± 0.0117 µg/g) (Figure 3a). The bar plots (Figure 3b) indicate that the highest average Cd concentration was observed during the transition season at VM-22-T (0.0025 ± 0.0004 µg/g), while the lowest average concentration was recorded during the rainy season at CB -23-R (0.0003 ± 0.0001 µg/g). Se (Figure 3c) indicates that the highest average concentrations were observed in 2022, especially at VM-22 transition season (0.00663 ± 0.001 µg/g) and SP-22-T (0.00583 ± 0.001 µg/g). In contrast, the lowest concentrations were found in 2023, particularly in the VM-23 transition season (0.00188 ± 0.0002 µg/g). With respect to Pb, a substantial increase in concentrations during 2023 was evident at both sites (CB and VM), and was particularly more pronounced during the VM rainy season (Figure 3d). The statistical analysis revealed significant differences in Hg, Se, Cd, and Pb concentrations in *P. viridis* between sites (CB and VM) and seasons for 2020, 2022, and 2023 (Tables S1–S4). For Hg, the mean concentrations in both sites were significantly higher in 2022 than 2023 during the transition epoch. Similar behavior was observed for Cd and Se. These peaks reflect a possible increase in anthropogenic inputs in 2022. The highest Se concentrations were observed in VM during the 2022 transition (VM-22-T); the concentrations in this period were significantly higher than those in other periods such as VM-22-R (*p* = 0.0008) and CB-20-R (*p* = 0.0001). As for Cd, the highest values were also recorded in VM-22-T, with statistically significant differences concerning VM-23-R (*p* = 0.0100) and VM-22-R (*p* = 0.0005). The box plot (Figure 4d) illustrates the distribution and variability of the data, highlighting significant differences between sites and years evaluated.

## 6. Discussion

### 6.1. Accumulation of Hg, Cd, Se, and Pb in the Perna viridis Soft Tissue

The city of Cartagena consists of a conglomerate of artificially connected islands. It features a canal system that directs water from the southern part of the Bay, where the Canal del Dique is located, to the north, where the Virgen marsh is situated. The water currents within the Bay vary seasonally, becoming more intense during the rainy season due to the influence of the Canal del Dique [66]. The two sites selected for this study are strategically positioned to observe processes occurring in the Bay and, subsequently, in the Virgen marsh. Additionally, the presence of the mussel *Perna viridis* in the Bay serves as an indicator of the behavior of the studied metals and metalloids due to the high number of organisms recorded in these areas, with individuals present across all life stages, including reproductively mature adults. This contrasts with other parts of the Bay, where their sizes do not exceed 20 mm (Pers. Obs.). Moreover, *P. viridis* is an efficient filter feeder, which enhances the absorption of bioavailable elements. This organism filters a wide range of suspended particles from the water column through water currents pumped via its siphon. Consequently, any changes in the water column can lead to variations in contaminant concentrations [14].

The changes in precipitation caused by the La Niña-El Niño phenomena resulted in increased turbidity and particulate matter between 2020 and 2022, followed by a decrease in 2023 [65,66]. However, a seasonal pattern was maintained, characterized by a dry period lasting until March, a minor rainy period between May and July with a decrease in precipitation until September (transition), and subsequently, a period of heavy rainfall lasting until December [66]. These variations led to important changes in the concentrations of Cd, Hg, Se, and Pb across the studied years. Thus, the bar graph illustrates notable fluctuations in average mercury concentrations by site and year. The highest concentrations were recorded during 2022, particularly at sites CB-2022 and VM-2022 in the transition season. These concentrations can be attributed to external events or factors that promoted the mobilization and accumulation of the metal in these systems. Such factors may include a rise in anthropogenic activities, such as mining, or an increase in maritime transport. Following the lifting of COVID-19 control and prevention measures, maritime transport notably increased, particularly in the Port of Cartagena [63]. In 2023, a contrasting pattern emerged, with lower concentrations observed primarily at site VM during the rainy season. This could be attributed to a reduction in contamination sources or an alteration in environmental conditions that limited mercury availability, such as greater dilution and transport to deeper areas or locations distant from the coast. Recent studies have reported a decline in mercury concentrations in sediments compared to levels observed during the 1980s [23]. These findings reflect the intricate distribution patterns of this element, likely influenced by local and temporal factors [66,67].

In detail, in 2022, a greater dispersion in the data and the presence of outliers was observed, particularly at sites CB-22 and CB-20, both in the rainy season. The scatter observed in the data suggests the occurrence of episodes of severe contamination or irregular inputs of mercury into the ecosystem, which has resulted in alterations to local conditions. In comparison, the 2023 chart demonstrates a narrower distribution, with lower scattering values and no significant outliers. This indicates a stabilization in mercury concentrations, which could be attributed to a reduction in pollutant inputs. In consideration of the dry season, the data presented in the graph demonstrate a reduction in the variability of Hg concentrations in comparison to the transition seasons. During the dry season, the boxes are narrower, indicating that mercury concentrations remain relatively constant over time. This may be because there is less water activity (such as rainfall or water flows) during this period, which reduces the mobilization of mercury in the environment and causes less variability in concentrations.

With respect to Cd concentrations, changes likely reflect an increase in anthropogenic sources of pollution during the period 2020–2022, possibly driven by industrial or port activities, exacerbated by the hydrological conditions typical of the transition season. In contrast, the lowest concentrations were recorded in 2023, particularly during the transition at VM-23-T (0.00011 µg Cd/g), indicating a reduction in pollutant loads or improvements in environmental management at the study sites. This greater scatter could reflect sporadic contamination episodes or localized discharges. The data from 2023 display less variability, particularly during the dry seasons, suggesting that cadmium concentrations became more consistent, possibly due to improved control of pollution sources or changes in environmental conditions [66].

High concentrations of Se were observed in 2022. These peaks highlight the role of transitional periods in exacerbating selenium accumulation, likely due to hydrodynamic changes that enhance pollutant deposition. These results are possibly linked to local discharge activities and seasonal hydrological dynamics, suggesting the presence of irregular contamination events and diffuse inputs of selenium. The lowest concentrations were found in 2023, particularly in the VM-23-T transition season. In contrast, the 2023 data show more homogeneous concentrations, particularly during the dry seasons, indicating fluctuating pollution levels and different sources among seasons and sites. This demonstrates the influence of environmental patterns and anthropogenic activities on the accumulation of metal concentrations in *P. viridis* [33,68].

The analysis of Pb concentrations in *P. viridis* between 2020 and 2023 reveals significant temporal and spatial variability between sites. In 2023, the CB site exhibited a wider range of Pb concentrations with higher maximum values, indicating more heterogeneous contamination. This could be related to sediment loading from the Canal del Dique, especially after the end of the La Niña phenomenon and the onset of El Niño, which likely increased sediment transport to CB. These Pb-enriched sediments, entrained by the changing hydrodynamic conditions, would have been bioavailable to *P. viridis*, resulting in higher concentrations at this site. In contrast, concentrations at VM during 2023 showed a more stable pattern with less variability.

The site VM showed relatively uniform Pb levels, suggesting a more consistent or localized source of contamination compared to CB. This stability could also indicate that sediment transport from the Canal del Dique had a less pronounced effect on VM in 2023, possibly due to distance from the canal site or changes in water flow dynamics. However, in 2022, VM recorded significantly higher Pb concentrations, particularly during the rainy season. The increased precipitation during this period likely resulted in higher sediment runoff from the Canal del Dique, bringing more Pb-enriched sediments into VM. This contrast highlights how seasonal and climatic factors can influence contamination levels, with the rainy season contributing to elevated concentrations at VM in 2022 but not in 2023, when sediment transport patterns may have been different. In 2020 and 2022, both sites showed lower Pb concentrations with reduced variability, reflecting more stable environmental conditions and less contamination pressure during these years. The results confirm the usefulness of *P. viridis* as a bioindicator. The high concentrations of Se, Cd, Pb and Hg in 2022, associated with human activities, underline the need for environmental strategies to reduce pollutant sources and continuous monitoring in Cartagena Bay.

### 6.2. Threshold Levels for Heavy Metal Contamination

The results of this study indicated that the concentrations of mercury, cadmium, and lead detected do not exceed the permissible limits for seafood and other food consumption established by Colombian or international standards (Table 5). However, frequent consumption of seafood with the recorded concentrations could lead to toxic effects due to bioaccumulation, particularly in vulnerable populations such as infants, the elderly, or individuals with preexisting health conditions that may be exacerbated by the presence of these metals in the body [13].

### 6.3. Concentration of Metals in P. viridis in Different Regions

The results highlight the significant impact of climatic patterns on the accumulation of metal concentrations in *Perna viridis*, consistent with findings from studies on other bivalve species conducted in earlier years (Table 6). Regarding regions where this species is native, Hg concentrations fell within the typical range reported in Malaysia and Indonesia. Cd concentrations were lower than those recorded in the same regions [46,67], while Pb concentrations were below the levels documented in highly impacted areas such as Japan [51]. For the Caribbean (Table 6), studies on metal concentrations in *Perna viridis* are limited. Those conducted in Venezuela [68], Jamaica [59], and Trinidad and Tobago [58] are outdated. Compared to these, our results showed that the concentrations recorded in *P. viridis* remained at low levels. Additionally, it was observed that the organism can reflect differences between sites and local conditions, highlighting its potential as a biomonitoring species. In Colombia (Table 6), no studies have been conducted on this mussel despite its presence being recorded since 2009 in sites with similar characteristics. This is the first study on metals in *Perna viridis* in Colombia and only the second in South America. Regarding Hg and Pb, concentrations remained below permissible levels established nationally and internationally. For Cd, a significant decrease in concentrations was identified in *P. viridis* compared to those reported for *Saccostrea* sp., another invasive species found in the Colombian Caribbean [75].

## 7. Practical Implications

The establishment of *Perna viridis* as an invasive species in the Colombian Caribbean poses complex ecological and environmental implications. On one hand, its colonization in Cartagena Bay and other areas highlights its adaptability and competitive advantages over native bivalves, such as *Crassostrea rhizophorae* and *Isognomon alatus*. These advantages, including high reproductive rates and broad tolerance to environmental fluctuations, have enabled it to displace native species, leading to changes in benthic community composition and potential disruptions in ecosystem functioning [20,45]. Such displacement could affect nutrient cycling, sediment stability, and trophic interactions in these marine environments. However, the ability of *P. viridis* to bioaccumulate pollutants offers an opportunity to leverage its presence for ecological monitoring, providing critical data on contaminant dynamics in areas impacted by industrial and urban activities [32,50].

From an environmental perspective, the established populations of *P. viridis* in Cartagena Bay have proven useful for tracking pollutants such as mercury, cadmium, lead, and selenium, which are frequently associated with the region’s industrial and maritime activities [62]. These insights are meaningful for identifying pollution hotspots and assessing the effectiveness of environmental management strategies in a region heavily influenced by mining, refinery operations, and port activities [32,62]. Its ability to thrive in variable environmental conditions makes it a reliable organism for biomonitoring in Cartagena Bay and other parts of the Colombian Caribbean. It is important that future research analyzes the different intrinsic and extrinsic factors that determine its performance as a biomonitor [48,49], considering the spatial variability and dynamics of pollutants not only in SPM but also in water and sediment. Thus, the potential of *P. viridis* can be integrated into environmental studies that support decision making in pollution control policies and programs, while balancing the ecological risks associated with its invasive nature [15,20].

## 8. Conclusions

This study addressed the presence of *Perna viridis* in the Colombian Caribbean as both an invasive species and a potential biomonitor for aquatic pollution. Its capacity to bioaccumulate contaminants like mercury, cadmium, lead, and selenium provides insights into pollutant dynamics in Cartagena Bay, a region heavily influenced by industrial and maritime activities. Seasonal and spatial variability in metal concentrations suggested the impact of hydrological and anthropogenic factors on contaminant distribution. While its invasive nature poses ecological risks, *P. viridis* could be used in environmental management strategies for pollution monitoring.

## Figures and Tables

**Table 1:** Parameters of the atomic absorption analysis with graphite furnace (GF-AAS).

Metal	Wavelength (nm)	Lamp Current (%)	Standards Concentration (μg/L)
Cd	228.8	70	3/5/8/10/15/20/30
Pb	217.0	80	5/10/25/50/100/150/220
Se	196.0	80	50/100/250/350/450/650

**Table 2:** Temperature programming of the graphite furnace atomic absorption instrument.

Steps	Temperature (°C)			Time (s)			Ramp (°C/s)			Gas flow (L/min)		
	Cd	Pb	Se	Cd	Pb	Se	Cd	Pb	Se	Cd	Pb	Se
1° Drying	110	110	110	30	30	40	10	10	20	0.1	0.2	0.1
2° Drying	125	120	120	30	10	20	10	10	10	0.1	0.1	0.1
Pyrolysis	550	900	1100	25	20	20	150	150	80	0.2	0.2	0.1
Atomization	1000	1350	2300	3	3	3	0	0	0	Off	Off	Off
Cleaning	2500	2500	2500	3	3	3	0	0	0	0.2	0.2	0.2

**Table 3:** Cd, Pb, Hg in suspended filtered material (mean ± SD µg/g d.w).

Season	Date	Sampling Site	Cd	Pb	Hg
Rainy	20 November 2020	VM	0.33 ± 0.001	25.66 ± 1.47	1.20 ± 0.24
	26 October 2020	CB	0.08 ± 0.05	10.94 ± 0.79	0.50 ± 0.05
Transition	29 June 2022	VM	0.13 ± 0.01	10.17 ± 0.29	0.32 ± 0.09
	6 July 2022	CB	0.18 ± 0.02	4.9 ± 0.40	0.28 ± 0.08
Rainy	3 November 2022	VM	0.11 ± 0.01	12.48 ± 8.93	0.18 ± 0.08
	10 November 2022	CB	0.13 ± 0.19	8.84 ± 5.58	0.50 ± 0.22
Dry	3 March 2023	VM	0.17 ± 0.13	22.68 ± 24.7	0.25 ± 0.14
	2 March 2023	CB	0.07 ± 0.00	4.94 ± 0.23	0.33 ± 0.14
Rainy	17 November 2023	VM	0.07 ± 0.01	7.23 ± 1.05	<LOQ
	22 November 2023	CB	0.07 ± 0.01	5.14 ± 0.35	<LOQ

**Table 4:** Cd, Pb, Hg, and Se in the soft tissue of *Perna viridis* (mean ± SD µg/g d.w.).

Season	Sampling Date	Sampling Site	Sampling Code	Cd	Hg	Pb	Se
Rainy	26 October 2020	CB	CB-20-R	0.0034 ± 0.0007	0.14 ± 0.02	0.34 ± 0.15	0.0080 ± 0.0009
Transition	29 June 2022	VM	VM-22-T	0.0013 ± 0.0001	0.20 ± 0.02	0.65 ± 0.17	0.0103 ± 0.0018
	6 July 2022	CB	CB-22-T	0.0039 ± 0.0007	0.21 ± 0.04	0.61 ± 0.19	0.0090 ± 0.0020
Rainy	3 November 2022	VM	VM-22-R	0.0006 ± 0.0001	0.15 ± 0.01	0.34 ± 0.12	0.0061 ± 0.0005
	10 November 2022	CB	CB-22-R	0.0028 ± 0.0012	0.11 ± 0.03	0.28 ± 0.19	0.0059 ± 0.0004
Dry	3 March 2023	VM	VM-23-D	0.0005 ± 0.0003	0.13 ± 0.02	0.09 ± 0.06	0.0038 ± 0.0003
	2 March 2023	CB	CB-23-D	0.0004 ± 0.0002	0.12 ± 0.01	0.05 ± 0.07	0.0038 ± 0.0005
Transition	10 July 2023	VM	VM-23-T	0.0002 ± 0.0001	0.10 ± 0.02	0.23 ± 0.09	0.0029 ± 0.0003
	11 July 2023	CB	CB-23-T	0.0004 ± 0.0002	0.08 ± 0.01	0.76 ± 0.49	0.0037 ± 0.0005
Rainy	21 November 2023	VM	VM-23-R	0.0001 ± 0.0000	0.04 ± 0.02	1.18 ± 0.32	0.0029 ± 0.0005
	7 November 2023	CB	CB-23-R	0.0003 ± 0.0001	0.11 ± 0.01	0.47 ± 0.33	0.0034 ± 0.0007
*p*-value ^1^				<0.001	<0.001	<0.001	<0.001

^1^ Kruskal–Wallis rank sum test.

**Table 5:** Permissible limits (µg/g) for Cd, Hg, Se, and Pb established by national and international authorities.

Law/Institution	Sample Evaluated	Permissible Limit Cd	Permissible Limit Pb	Permissible Limit Hg	Reference
U.S. Food and Drug Administration (FDA)	Oysters	---	---	0.25	[69]
Food Sanitary Regulation, Chile	Shellfish	0.5	5	0.5	[70]
Sanitary Regulation, Colombia	Food Quality	0.05	1.5	0.5	[71]
NOAA Mussel Watch Program (U.S.)	Mussels	4		1	[72]
European Union	Shellfish	1	1.5	0.5	[73]
World Health Organization	Food	10	5–30		[74]

**Table 6:** Measurements of metals in *Perna viridis* and other bivalves.

Site/Sampling Season	Species	Metal	Metal Concentration	Reference
Japan: January–March; July–September, 2019	*P. viridis*	Pb	1.76–3.28	[51]
Malasia: March–August, 2018	*P. viridis*	Pb, Cd, Hg	Pb 0.06–0.78; Cd 0.16–2.12; Hg 0.05–0.06	[67]
Indonesia: July–October, 2019	*P. viridis*	Pb, Cd, Hg	Pb 0.06–3.498; Cd 0.005–2.47; Hg 0.001–0.156	[46]
Trinidad and Venezuela: June–December, 1999.	*P. viridis*	Cd, Hg	Cd 0.02–0.61; Hg	[58]
Jamaica: March, 2001	*P. viridis*	Cd, Pb	Cd 17.00–60.00; Pb 0.20–0.40	[59]
Venezuela: Rain drought, 2012	*P. viridis*	Cd, Pb	Cd 1.23 ± 0.42; Pb 0.19 ± 0.47	[68]
Cartagena Colombia, November 1980	*Crassotrea rhizophorae* *Isognomon alatus*	Cd, Pb Cd, Pb	2.51–15.90; 1.26–5.13 0.80–15.60; 0.75–3.16	[76]
Cartagena Colombia, September/2012 May/2013	*Donax denticulatus*	Hg, Pb, Cd	Hg 0.006; Pb 0.060; Cd 0.040	[77]
Cartagena Colombia, October 2012 and March 2013	*Crassostrea rhizophora*	Cd, Hg, Pb	Cd 2.54–28.03; Hg 0.03–0.09; Pb 0.15–0.60	[33]
Cartagena Colombia, October 2012 March 2013	*Saccostrea sp.*	Cd, Hg, Pb	Cd 3.43–15.88; Hg 0.04–0.09, Pb 0.15–0.75	[1]
Cartagena Colombia, October 2020 July, Nov. 2022 March, July, Nov. 2023	*P. viridis*	Cd, Hg, Se, Pb	Cd 0.00173–0.00311; Hg 0.0725–0.141; Se 0.00408–0.00619; Pb 0.121–0.474 Cd 0.000332–0.00332; Hg 0.0607–0.181; Se 00354–0.00851; Pb 0.0970–0.587 Cd 0.0000249–0.000641; Hg 0.0194–0.0976; Se 0.00147–0.00295; Pb 0.00–1.245	This study

## Data Availability

Dataset available on reasonable request.

## Supplementary Materials

The following supporting information can be downloaded at: https://www.mdpi.com/article/10.3390/toxics13020077/s1, Table S1: Pairwise multiple comparisons of the ranked data for year, season, and sampling sites for Cadmium (Cd) using Dunn’s test with Bonferroni correction; Table S2: Pairwise multiple comparisons of the ranked data for year, season, and sampling sites for Lead (Pb) using Dunn’s test with Bonferroni correction; Table S3: Pairwise multiple comparisons of the ranked data for year, season, and sampling sites for Mercury (Hg) using Dunn’s test with Bonferroni correction; Table S4: Pairwise multiple comparisons of the ranked data for year, season, and sampling sites for Selenium (Se) using Dunn’s test with Bonferroni correction.
